# Metastatic Gas Gangrene

**Published:** 1917-12

**Authors:** 


***Metastatic Gas Gangrene.*** By J. N. J. Hartley, Capt. R. A. M. C. (T. C.). Abstract. from the *British Medical Journal*, April 14, 1917.

H. has observed five cases in which micro-organisms of gas gangrene have gained entrance to the blood stream and have produced lesions in distant parts of the body. The author gives full details of the five cases, three of which ended in recovery. In all cases in which a bacteriological examination was made, B. aerogenes capsulatus was the causal organism of the metastatic infection. In four cases the secondary lesions occurred in the buttocks. The author agrees with Taylor that this fact was problably due to the posture of the patient. In two cases eusol was injected intravenously, but the author cannot declare that this was necessarily the cause of recovery, because a third case in which eusol was not used developed satisfactorily also. In this last case, although there was systemic infection there was an absence of marked toxemic symptoms. In one case extensive gas gangrene in the right buttock and thigh occurred five days after amputation of the right arm. In another case the metastatic gangrene occurred a week after the amputation of gangrenous feet. In these last two cases only slight incisions were necessary for the secondary lesions, the infection was localized, and the infected muscles sloughed spontaneously. The author believes the injections of eusol in these two cases tended to retard the infection, and so helped the patient to develop resistance to meet the secondary infection.

